# Effect of Eel Cookie Supplementation on the Hemoglobin Status of Pregnant Women with Anaemia: A Pilot Study

**DOI:** 10.1155/2022/3919613

**Published:** 2022-01-24

**Authors:** Dewi Marhaeni Diah Herawati, Deni Kurniadi Sunjaya, Lailia Fathul Janah, Nita Arisanti, Hadi Susiarno

**Affiliations:** ^1^Department of Public Health, Division of Medical Nutrition, Faculty of Medicine, Universitas Padjadjaran, Indonesia; ^2^Department of Public Health, Division of Public Health, Faculty of Medicine, Universitas Padjadjaran, Indonesia; ^3^Sukabumi District of Health Office, Indonesia; ^4^Department of Obstetrics and Gynecology, Faculty of Medicine, Universitas Padjadjaran, Indonesia

## Abstract

**Background:**

Anaemia in pregnancy is a major global health problem. Iron and folic acid (IFAS) and animal-based supplement consumption could improve the hemoglobin status of pregnant women. This study is aimed at determining the effect of eel cookie supplementation on hemoglobin levels of pregnant women.

**Methods:**

This pilot study with pretest–posttest design was conducted in Tamanjaya Public Health Center, Geopark Ciletuh, Sukabumi District, West Java Province, Indonesia. A total of 40 voluntary pregnant women were divided into two groups: an intervention group that received 11 pieces of eel cookies and control group that received the same number of cookies without eel. The women's hemoglobin level was analysed using *t*-test, Mann-Whitney, Wilcoxon, and analysis of covariance tests.

**Results:**

After consuming the eel cookies for 1 month, there was a significantly greater increase in the hemoglobin levels in the intervention group than those in the control group, which were 1.69 and 0.69 g/dL, respectively. Improvement in percentage of mean hemoglobin was higher in the intervention group than in the control group, which was 17.21% and 6.53%, respectively.

**Conclusions:**

Supplementation with eel cookies and IFAS for 1 month could improve the hemoglobin status in pregnant women with anaemia.

## 1. Introduction

Anaemia in pregnancy is a major global health problem, with a prevalence of 41.8% [1]; the highest prevalence is in Africa, at 61.3%, and it is 52.5% in Southeast Asia and [2] 37.1% in Indonesia [3]. The aetiology of anaemia is multifactorial, but the main cause is iron deficiency [2, 4]. If anaemia is not treated properly, it could result in maternal and foetal mortality [5] and premature birth [6]. According to WHO, pregnant women are considered anaemic if their hemoglobin levels during the 1^st^ and 3^rd^ trimester are lower than 110 g/dL [2]. During pregnancy, women should consume iron supplements to build sufficient iron reserves to prevent iron deficiency [7]. The WHO recommends supplementation with 30-60 mg of iron and 400 *μ*g (0.4 mg) of folic acid daily throughout pregnancy, to prevent anaemia [8].

Iron and folic acid supplementation (IFAS) for pregnant women could prevent anaemia in the 3^rd^ trimester [9] and improve birth outcomes [10]. IFAS has proven to be beneficial in reducing the risk of postpartum haemorrhage, premature birth, and low foetal birth weight, which could result in maternal and infant mortality [11]. Iron absorption, formation, and use in the body are regulated by a strict iron homeostatic regulation mainly through the mediation of absorption and recycling [12].

Anaemia prevention program in Indonesia is aimed at providing IFAS for 90 days. This program was not in accordance with WHO recommendations, which states that supplementation should be provided throughout pregnancy. The Indonesian government's efforts to increase IFAS consumption in pregnant women were through free supplementation programs. Despite these efforts, the prevalence of anaemia in pregnant women was still quite high. Some of the contributing factors include poor IFAS consumption compliance [13] and inappropriate IFAS consumption.

IFAS consumption compliance in Indonesia was only 52.9% [14], which was similar to that in Kenya (55.1%) [15]. Poor compliance in consuming IFAS could affect maternal and infant health [16]. Poor adherence was caused by the side effects of IFAS consumption in the form of gastrointestinal disorders, such as nausea, vomiting, diarrhoea, constipation, and abdominal pain [17].

In addition, pregnant women often consume IFAS with tea or milk instead of water. The tea could interfere iron absorption and cause iron deficiency anaemia if consumed in large quantities [18]. It is the polyphenol content in tea that interferes with heme and nonheme iron absorption [19]. Tannins present in tea also caused inhibition of iron mineral bioavailability when consumed in large quantities [20] but had no effect on changing iron status [21] and iron deficiency incidence [22].

Pregnant women with anaemia were recommended to consume extra protein, vitamin C, and vitamin A (VA) to promote IFAS absorption. A high-protein diet could enhance nonheme iron absorption [23]. Vitamin C supports iron absorption [24], whereas VA plays an important role in iron metabolism [25]. Researchers made cookies made of eel liver and corn flour. These eel cookies had high protein, iron, and VA. The purpose of this study was to compare the effects of IFAS consumption with eel cookies and with cookies without eel on the hemoglobin status of pregnant women with anaemia.

## 2. Materials and Methods

### 2.1. Study Design

The design of this pilot study was experimental with pre- and posttest. The study was conducted in Tamanjaya Public Health Center located in Geopark Ciletuh, Sukabumi District in West Java. We selected pregnant women who had mild anaemia with hemoglobin levels of 8-11 grams%, in the 2^nd^ to 3^rd^ trimester of pregnancy, and who were willing to consume eel cookies for 1 month. Pregnant women with chronic underlying diseases and allergies were excluded.

### 2.2. Study Participant

**Figure 1:**
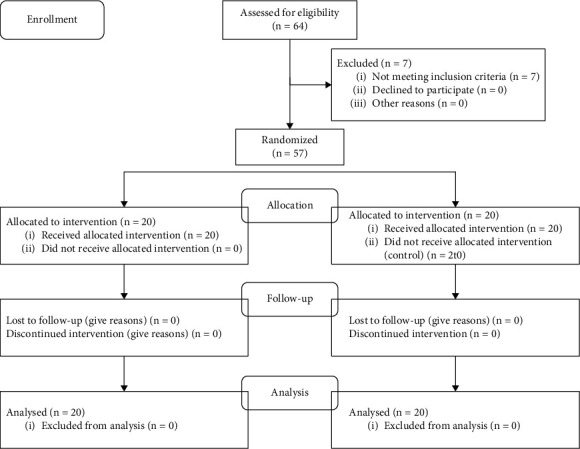
Flow diagram.

The number of anaemic pregnant women obtained at the time of screening was 64 people. However, 7 pregnant women were not willing to be respondents, so there were 57 people who were willing. From 57 people, 40 people were taken randomly by taking all the names of pregnant women. The name that comes out first will enter the control group, and the second will enter the intervention group and so on until each group consists of 20 people (Figure 1).

Sample size was determined for comparison of two proportions with 80% power and 5% significance level, alpha < 0.05 one-sided. The assumed standard deviation is 12, obtained from the research results of Briawan et al. [26]. Based on the calculation, the number of samples per group is 17. To avoid miss follow-up, the number of samples is increased by 10% to 20 per group.

This research was conducted in 4 villages in Tamanjaya Public Health Center, Sukabumi Regency. The four villages are Tamanjaya, Mekarsakti, Ciwaru, and Mandrajaya. All participants did not know which one was the control group and which one was the intervention group, because the biscuits have the same shape and colour. Supplementation with 60 mg iron tablets and 250 mcg folic acid daily was mandatory in both groups. The chemical property of iron is ferrous fumarate ion. For the intervention group, eel biscuits were added, while the control group was given biscuits without eel. All participants consumed 11 biscuits (100 g) every day for 1 month.

### 2.3. Ethical Approval

This study fulfilled the principles of the Helsinski's declaration and received ethical clearance from the Ethics Committee of the Faculty of Medicine of Padjadjaran University, No. 106/UN6.C.10/PN.2017. Informed consent was obtained from all subjects before participating in this study.

### 2.4. Data Collection and Measurements

All participants were subjected to a nutritional assessment (i.e., anthropometric measurements and dietary assessments) and the determination of hemoglobin levels. Anthropometric measurements including body weight (BW), height, midupper arm circumference (MUAC), and body mass index (BMI), as well as hemoglobin levels, were assessed before the intervention and 2 and 4 weeks after the intervention. Hemoglobin estimation was performed using the hemoglobin digital system (quick hemoglobin testing system). The BW was measured using the Camry digital scale, while height was measured using a microtoise and MUAC using measurement tapes. A simple 24-hour dietary recall was conducted before and after the intervention by the research team assisted by nutritionists to determine if there was a difference in dietary intake between the two groups. Anthropometric measurements and dietary assessment were carried out by enumerators (nutritionists), while hemoglobin level determination by midwives. Monitoring of iron consumption and cookies is carried out by families every day and by cadres every 2 days for 1 month. Meanwhile, village midwives and nutrition officers supervise every 3 days for 1 month. Monitoring uses forms to see compliance and side effects. During the study, none of the pregnant women complained of side effects such as allergies and diarrhea.

### 2.5. Supplementary Cookies

The eel cookies were made using eel liver flour, corn flour, cocoa powder, nuts, and other additional ingredients. Meanwhile, the cookies for the control group were made using the same ingredients, except for the eel liver flour. The nutrient composition in both sets of cookies is shown in Table 1. Assessment of nutritional content such as macronutrients and micronutrients in both cookies was carried out through proximate, vitamin, and mineral tests. All tests were carried out at the Saraswanti laboratory, Bogor, including testing for proximate, vitamins, minerals, metals, and microbiology. Nutritional content of cookies was 100 g. All test results meet the qualifications of the Indonesian National Standard (SNI) for cookies (Table 1).

### 2.6. Statistical Analysis

The data was analysed using the SPSS software (version 20). Univariate analysis was performed using chi-squared and Mann-Whitney tests to determine differences among the participants' demographic characteristics. For screening for normality of data distribution, Shapiro-Wilk was used, where it is normal if the value was greater than 0.05.

Bivariate analysis on anthropometric data was conducted using *t*-test (for normal data distribution, e.g., MUAC), Fisher exact test (for categorical data), and Mann-Whitney test (for not normal numerical data distribution, e.g., BMI). Nutritional intake was analysed using *t*-test and Mann-Whitney test, while hemoglobin levels were analysed using *t*-test, Mann-Whitney (for independent, numerical with data distribution was not normal, e.g., hemoglobin between intervention and control groups), Wilcoxon test (for pair but not normal numerical data distribution, e.g., hemoglobin pre and post), and analysis of covariance. Statistical significance was set at a *p* value < 0.05.

## 3. Results and Discussion

### 3.1. Results

Comparisons of data on demographic characteristics, including pregnancy risk, parity, parents' education, and occupations, between the intervention and control groups did not show any significant difference (Table 2). Most of the women participated in this study and their husbands had low education levels. The husbands worked mostly as entrepreneurs, and the women were unemployed.

There was no significant difference in BW, MUAC, height, and BMI between the two groups, as indicated in Table 3. Both groups were relatively homogeneous.

The protein intake in posttest was significantly different between the intervention and control groups (*p* = 0.028). Other nutritional intake, including VA, protein in pretest, iron, vitamin C, calories, and energy, was not significantly different between the two groups (Table 4).

The compliance rates of cookie consumption were similar in both groups (Table 5). However, four people in the control group consumed only 4-5 pieces of cookies per day.

**Figure 2:**
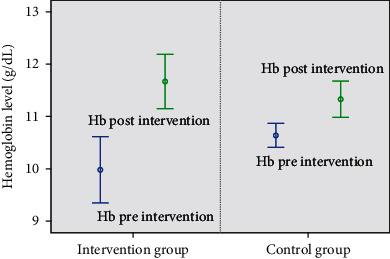
Changes in the percentage of mean of hemoglobin in both groups.

As shown in Table 6, after consuming the cookies for 1 month, there was a significantly greater increase in the hemoglobin levels in the intervention group than those in the control group (*p* < 0.001), which were 1.69 and 0.69 g/dL, respectively. Additionally, the adjusted percentage of mean Hb in the intervention group showed a higher improvement than that in the control group (17.26% and 6.49%, respectively). The eel cookie supplementation had a greater impact on hemoglobin levels than the cookies without eel (Figure 2).

## 4. Discussion

The results of this study indicated that consumption of eel cookies and IFAS for 1 month could increase the mean hemoglobin levels of pregnant women by 1.69 g/dL, while in the control group, the increase was only 0.69 g/dL. Based on the statistical analysis, there was a significant difference in the protein intake as well. The higher protein intake in the intervention group could be the reason for the greater improvement in hemoglobin status. This was in accordance with the findings of Anwar et al.'s study, which revealed that pregnant women who received protein and iron-fortified crackers had a higher increase in hemoglobin levels than pregnant women who received only iron-fortified crackers [27]. The content of fish protein in the cookies, as well as beef protein according to Geissler and Singh, was able to improve the absorption of nonheme iron, resulting in increased hemoglobin levels [27, 28]. The protein content in eel cookies was higher than that in the usual fish protein crackers.

Eel cookies also had a high VA content, which is essential in the metabolism/absorption of iron. This result was in line with Hurell and Egli findings, which stated that simultaneous consumption of iron and VA was more effective in increasing hemoglobin levels than iron consumption alone [29]. A study by Muslimatun et al. also supported this results, which showed that the provision of VA and IFAS supplementation had a greater impact on increasing hemoglobin levels than IFAS supplementation alone [30]. According to Zimmerman et al., VA enhanced the absorption and/or metabolism of iron [25]. The iron level in the human body is regulated mainly by absorption, and there is no physiological mechanism for iron excretion. VA affects iron metabolism by promoting erythropoiesis and the release of iron from ferritin stores [31].

Heme iron is the most bioavailable form, with an absorption rate of approximately 11-22% compared to nonheme iron, with a rate of 1%-7% [32]. Animal food products are rich in protein, iron, iodine, zinc, and vitamin B12. Although red meat consumption could increase serum ferritin by 36% more than any other animal foods [33], its high cost makes it undesirable as a supplement, especially in developing countries. Hence, animal food supplements made from fish were preferable, such as locally available eel for pregnant women in Sukabumi District, to provide extra heme iron.

The compliance rate of IFAS consumption was similar in the two groups, while the compliance rate of cookie consumption in the intervention group was slightly higher than that in the control group (86% vs. 82%, respectively). However, roughly 18% of the respondents consumed only 4-5 pieces of cookies, which was less than half of the recommended portion. These results were different from the studies conducted on stunted children, in which the intervention group that received eel cookies showed a greater compliance rate than the control group. However, the IFAS consumption compliance in both groups was also similar, which was 92% [34].

This study had several limitations, including the small sample size because it was a pilot study. This study estimated only hemoglobin levels, not accompanied by ferritin tests. In the future, a cohort study with a large sample size is suggested to assess the foetal outcomes of this supplementation.

## 5. Conclusions

Eel cookie supplementation and IFAS consumption for 1 month could improve the hemoglobin status of pregnant women with anaemia. The intervention group had a greater increase in mean hemoglobin levels than the control group.

## Figures and Tables

**Table 1:** Comparison of eel cookies and cookies without eel composition/100 g.

Parameter	Eel cookies	Cookies without eel
Energy (kcal)	349.1	320
Carbohydrate (g)	30.2	45
Fats (g)	20.7	12
Protein (g)	10.5	8
Iron (mg/ppm)	33	17
Vitamin A (mcg)	793.3	258

**Table 2:** Comparison of demographic characteristics of intervention and control groups.

No.	Variable	Groups		*p* value
		Intervention (*n* = 20)	Control (*n* = 20)	
1.	Pregnancy risk			0.288^∗^
	Low risk	13	16	
	High risk	7	4	
2.	Parity			0.240^∗^
	Primipara	4	7	
	Multipara	14	13	
	Grande multipara	2	0	
3.	Mother's education			0.786^∗^
	Elementary school	9	8	
	Junior high school	8	10	
	Senior high school	3	2	
4.	Mother's occupation			1.000^∗∗^
	Unemployed	18	19	
	Employed	2	1	
5.	Husband's education			0.762^∗^
	Elementary school	9	10	
	Junior high school	5	6	
	Senior high school	6	4	
6.	Husband's occupation			0.461^∗^
	Civil servant/teachers	0	2	
	Private employees	2	3	
	Entrepreneurs	11	10	
	Farmer/laborer	7	5	
7.	Family income			0.809^∗^
	<1 million	6	5	
	1-3 million	13	13	
	>3-5 million	1	2	
8.	History of antenatal care			
	1^st^ trimester	1 (1-3)^∗^	2 (0-4)	0.277^∗^
	2^nd^ trimester	3 (1-5)^∗^	3 (1-4)	0.989^∗^
	3^rd^ trimester	0 (0-2)^∗^	1 (0-5)	0.301 ^∗∗∗^

Data are expressed as median and range; ^∗^chi-square; ^∗∗^Fisher; ^∗∗∗^Mann-Whitney; significance level applied at *p* < 0.05.

**Table 3:** Comparison of anthropometric measurements in both groups.

No.	Variable	Groups		*p* value
		Intervention (*n* = 20)	Control (*n* = 20)	
1.	BW pre (kg)	55.2 (10.4) ^∗^	54.51 (11.31)	0.841^∗∗^
2.	BW post (kg)	61.8 (9.8)^∗^	61.23 (12.01)	0.873^∗∗^
3.	MUAC pre (cm)	24.7 (2.04)^∗^	25.6 (3.39)	0.698^∗∗∗^
4.	MUAC post (cm)	26.7 (1.8)^∗^	26.7 (3.83)	0.231^∗∗∗^
5.	Height (cm)	154.6 (4.8)^∗^	154.4 (4.7)	0.894^∗∗^
6.	BMI pre	23.06 (3.9)^∗^	22.92 (4.94)	0.718^∗∗∗^
7.	BMI post	25.8 (3.6)^∗^	25.7 (4.96)	0.926^∗∗∗^

^∗^Data are expressed as the mean and standard deviation; ^∗∗^*t*-test; ^∗∗∗^Mann-Whitney; significance level applied at *p* < 0.05.

**Table 4:** Vitamin A, protein, iron, vitamin C, calorie, and energy intake in both groups.

No.	Variable	Groups		*p* value
		Intervention (*n* = 20)	Control (*n* = 20)	
1.	Vitamin A			
	Pretest	777 (231–3093)	789 (256-1500)	0.355^∗∗∗^
	Posttest	900 (355-2685)	1002 (351-1639)	0.841^∗∗∗^
2.	Vitamin C			
	Pretest	27.7 (6-187.5)	39.05 (1.80-257.4)	0.738^∗∗∗^
	Posttest	29.5 (2.50-97)	29.3 (5.05-111.6)	0.779^∗∗∗^
3	Iron			
	Pretest	8.4 (3.4-18)	8.50 (2.0-76.10)	0.738^∗∗∗^
	Posttest	15.5 (4.6-54)	10.7 (4.90-22.70)	0.096^∗∗∗^
4	Calorie			
	Pretest	1264 (659-3543)	1373 (854-2956)	0.947^∗∗^
	Posttest	1399.5 (857-2220)	1464.5 (981-2141)	0.921^∗∗^
5	Protein			
	Pretest	48.50 (25-96)	34.50 (23-90)	0.76^∗∗^
	Posttest	58.50 (38-87)	42.50 (26-90)	0.028^∗∗^

Data are expressed as median and range; ^∗∗^independent *t*-test; ^∗∗∗^Mann-Whitney; significance level applied at *p* < 0.05.

**Table 5:** Compliance rate of IFAS and cookie consumption in both groups (%).

Cookie consumption	Groups	
	Intervention (*n* = 20)	Control (*n* = 20)
Not consumed	-	-
1–3 pieces	-	-
4–5 pieces	-	18%
6–7 pieces	-	-
8–10 pieces	14%	-
11 pieces	86%	82%
IFAS consumption	92%	92%

**Table 6:** Comparison of hemoglobin levels in both groups.

No.	Hb (g/dL)	Groups		*p* value
		Intervention (*n* = 20)	Control (*n* = 20)	
1.	Pretest			
	*Χ* (SD)	9.98 (0.63)	10.64 (0.23)	0.001^∗∗^
	Median	10.2	10.7	
	Range	8.3-10.7	10.2-10.9	
2.	Posttest			0.019^∗^
	*Χ* (SD)	11.67 (0.52)	11.33 (0.35)	
	Median	11.75	11.3	
	Range	10.5-13.00	10.9-12.0	
*p* value		*p* < 0.001^∗∗∗^	*p* < 0.001^∗∗∗^	
% mean Hb changes		17.21%	6.53%	<0.001^∗^
After adjusted protein intake		17.26%	6.49%	<0.001^∗∗∗∗^

Data are expressed as mean (*X*) and standard deviation (SD) and median and range; ^∗^*t*-test; ^∗∗^Mann-Whitney; ^∗∗∗^Wilcoxon; ^∗∗∗∗^ANCOVA; significance level applied at *p* < 0.05.

## Data Availability

The data used to support the study are available from the corresponding author on request.
